# Supplementary material for: Social and endogenous infant vocalizations
Source: PLoS One. 2020 Aug 5;15(8):e0224956. doi: 10.1371/journal.pone.0224956 (PMC7406057; doi:10.1371/journal.pone.0224956)
Supplement: Attachment — Submitted filename: Response to Reviewers.docx [file pone.0224956.s002.docx]

Social and endogenous infant vocalizations

Long, H. L., Bowman, D., Yoo, J., Burkhardt-Reed, M. M., Bene, E. R., & Oller, D. K.

**Rebuttal letter: Response to reviewers**

Links for each reviewer:

Response to Editor…2

Response to Reviewer 1: Cristia…8

Response to Reviewer 2: Gratier…12

References…20

# Response to Editor

PONE-D-19-29493
Social and non-social functions of infant vocalizations
PLOS ONE

Dear Ms. Long,

Thank you for submitting your manuscript to PLOS ONE. After careful consideration, we feel that it has merit but does not fully meet PLOS ONE’s publication criteria as it currently stands. Therefore, we invite you to submit a revised version of the manuscript that addresses the points raised during the review process.

As you will read in the attached reviews, both reviewers support the publication of the manuscript. Yet they both have some questions and comments and concerns about the paper which should be addressed in the revision.

These can be summarised as follows:

1) Background literature and theoretical assumptions. Both reviewers felt that the paper takes a somewhat narrow point of departure. Reviewer 1 for example suggests a more comprehensive literature review in the introduction. Along the same lines Reviewer 2 suggests that the authors adopt a rather narrow theoretical view. Both reviewers suggest that, both in the introduction and the discussion, there is a need to acknowledge other existing theoretical approaches and different interpretations of the findings. **On rereading, we agree that the perspective was too narrow, and that in particular, there was need for more nuance in the rationale about prior authors’ perspectives. We have revised in an attempt to provide a more balanced view, with many additional references. Various of the comments, especially from Gratier, make clear that our presentation of the idea of endogenously produced (and non-socially directed) infant vocalization, along with fitness signaling as a proposed selection pressure for it, was not elaborate enough, and in some ways was not optimally targeted. In particular we have reformulated for a more positive rationale, emphasizing the need for more attention to the endogenous vocalizations of human infants, but reducing the extent to which the rationale could be read as a critique of prior emphasis on vocal interaction—such a critique is unnecessary and so in large measure it has been eliminated or at least modified to reflect a more positive viewpoint. Also our prior writing seems to have left open interpretations about the motivations for protophone production that we do not intend and to have failed to imply interpretations we would have specified had we written a longer paper regarding the empirical findings. Thoughts about these comments have inspired rethinking on our part of the optimal rationale and interpretations. For example, the revision will clarify that we do not think of infant protophone production as “a residual behavior”. We seek also in the revision to avoid wording that might imply infant sound-making consists of “mere beeps of a survival instinct”. Although this single article may not provide space to be entirely explicit about our interpretations, we have modified the manuscript to try to clarify as much as possible and to incorporate an improved perspective. And we have formulated an extensive clarifying statement that is included in the new Supplementary Information (SI) as Appendix D. This addition presents an integrated perspective on the theoretical interpretations of protophone production as having been naturally selected for fitness signaling.**

2) Concerns with the Survey data. Both reviewers raised some concerns about the setup of the study and the contribution of its findings to the paper. One of the reviewers suggests minimising or excluding this study. They have provided questions and comments that should be addressed both in the manuscript as well as in the reply to reviewers. **We have opted to minimize the emphasis by moving the survey data and discussion to SI, offering more detailed rationale and description of methods for the survey there.**

3) Some questions and comments on data coding, clarification of selection criteria and further comments on the analysis (such as e.g. grouping ages instead of using age as a continuous variable). There are also some comments on potential additional analyses. **We supply both an additional analysis with age as a continuous variable and further clarifications on how the segments for analysis were selected and coded.**

4) Data availability. Review 1 commented on potentially making available annotated data if the raw data cannot be made available. Could the authors please also respond to this comment? **De-identified coding data is now supplied. We have posted it to Open Science Framework and, as instructed, include in the cover letter and on the cover page of the revised manuscript the relevant locating information.**

I believe that the reviewers have provided constructive comments which will allow the authors to adapt the manuscript and respond.

We would appreciate receiving your revised manuscript by May 22 2020 11:59PM. When you are ready to submit your revision, log on to <https://www.editorialmanager.com/pone/> and select the 'Submissions Needing Revision' folder to locate your manuscript file.

If you would like to make changes to your financial disclosure, please include your updated statement in your cover letter.

To enhance the reproducibility of your results, we recommend that if applicable you deposit your laboratory protocols in protocols.io, where a protocol can be assigned its own identifier (DOI) such that it can be cited independently in the future. For instructions see: <http://journals.plos.org/plosone/s/submission-guidelines#loc-laboratory-protocols> **We supply additional detail in the revision on how the data for the present study were selected and coded newly for this particular effort, reporting the method even more specifically than in the original submission. In particular we specify all the coding categories that were used, so there is no additional relevant coding protocol information. The method should be replicable based on these specifications. For years we have also maintained a site (babyvoc.org) that allows individuals to experience brief training on vocal coding for infants in the first year, although it does not specify the coding categories detailed in the revision.**

Please include the following items when submitting your revised manuscript:

- A rebuttal letter that responds to each point raised by the academic editor and reviewer(s). This letter should be uploaded as separate file and labeled 'Response to Reviewers'.
- A marked-up copy of your manuscript that highlights changes made to the original version. This file should be uploaded as separate file and labeled 'Revised Manuscript with Track Changes'.
- An unmarked version of your revised paper without tracked changes. This file should be uploaded as separate file and labeled 'Manuscript'.

Please note while forming your response, if your article is accepted, you may have the opportunity to make the peer review history publicly available. The record will include editor decision letters (with reviews) and your responses to reviewer comments. If eligible, we will contact you to opt in or out.

We look forward to receiving your revised manuscript.

Kind regards,

Iris Nomikou, Ph.D.
Academic Editor
PLOS ONE

Journal Requirements:

When submitting your revision, we need you to address these additional requirements.

1. Please ensure that your manuscript meets PLOS ONE's style requirements, including those for file naming. The PLOS ONE style templates can be found at
   <http://www.journals.plos.org/plosone/s/file?id=wjVg/PLOSOne_formatting_sample_main_body.pdf> and <http://www.journals.plos.org/plosone/s/file?id=ba62/PLOSOne_formatting_sample_title_authors_affiliations.pdf>

**The paper has been reformatted to meet PLOS ONE’s style requirements.**

2. In your Data Availability statement, you have not specified where the minimal data set underlying the results described in your manuscript can be found. PLOS defines a study's minimal data set as the underlying data used to reach the conclusions drawn in the manuscript and any additional data required to replicate the reported study findings in their entirety. All PLOS journals require that the minimal data set be made fully available. For more information about our data policy, please see <http://journals.plos.org/plosone/s/data-availability>.

**We have uploaded the dataset used in analysis to Open Science Framework and have revised our Data Availability statement on the first page of the revised manuscript.**

Upon re-submitting your revised manuscript, please upload your study’s minimal underlying data set as either Supporting Information files or to a stable, public repository and include the relevant URLs, DOIs, or accession numbers within your revised cover letter. For a list of acceptable repositories, please see <http://journals.plos.org/plosone/s/data-availability#loc-recommended-repositories>. Any potentially identifying patient information must be fully anonymized. **Posted to OSF:** <https://osf.io/hb834/>

Important: If there are ethical or legal restrictions to sharing your data publicly, please explain these restrictions in detail. Please see our guidelines for more information on what we consider unacceptable restrictions to publicly sharing data: <http://journals.plos.org/plosone/s/data-availability#loc-unacceptable-data-access-restrictions>. Note that it is not acceptable for the authors to be the sole named individuals responsible for ensuring data access.

We will update your Data Availability statement to reflect the information you provide in your cover letter.

Additional Editor Comments (if provided):

Dear authors,

I would like to apologise for the long wait. I took a lot of time to secure the reviewers. It was my mistake to grant reviewers extension after extension in hope that they would submit their reviews. This was because I really wanted to secure the most appropriate reviewers for your paper, which I think in the end I did.

As you will see both reviewers are really positive and so I hope that the second round of the review will run smoothly.

Best wishes,

Iris Nomikou


[Note: HTML markup is below. Please do not edit.]

Reviewers' comments:

Reviewer's Responses to Questions

Comments to the Author

1. Is the manuscript technically sound, and do the data support the conclusions?

The manuscript must describe a technically sound piece of scientific research with data that supports the conclusions. Experiments must have been conducted rigorously, with appropriate controls, replication, and sample sizes. The conclusions must be drawn appropriately based on the data presented.

Reviewer #1: Partly

Reviewer #2: Yes

2. Has the statistical analysis been performed appropriately and rigorously?

Reviewer #1: Yes

Reviewer #2: Yes

3. Have the authors made all data underlying the findings in their manuscript fully available?

The [PLOS Data policy](http://www.plosone.org/static/policies.action#sharing) requires authors to make all data underlying the findings described in their manuscript fully available without restriction, with rare exception (please refer to the Data Availability Statement in the manuscript PDF file). The data should be provided as part of the manuscript or its supporting information, or deposited to a public repository. For example, in addition to summary statistics, the data points behind means, medians and variance measures should be available. If there are restrictions on publicly sharing data—e.g. participant privacy or use of data from a third party—those must be specified.

Reviewer #1: No

Reviewer #2: No

4. Is the manuscript presented in an intelligible fashion and written in standard English?

PLOS ONE does not copyedit accepted manuscripts, so the language in submitted articles must be clear, correct, and unambiguous. Any typographical or grammatical errors should be corrected at revision, so please note any specific errors here.

Reviewer #1: Yes

Reviewer #2: Yes

5. Review Comments to the Author

Please use the space provided to explain your answers to the questions above. You may also include additional comments for the author, including concerns about dual publication, research ethics, or publication ethics. (Please upload your review as an attachment if it exceeds 20,000 characters)

# Response to Reviewer 1: Cristia

Reviewer #1: The manuscript is technically sound but two key changes are necessary for the conclusions to be directly tested/supported.

First, it is stated that a social interpretation is predominant in the literature, but only one citation is provided. I recommend a small but unbiased literature review (perhaps sampling 50 papers from pubmed with search terms "infant vocalization protophones"), to make sure that "research on human infancy [emphasizes] the dyad to the near exclusion of interest in the independent infant as an agent in its development". **About the single citation, the original statement includes references 1-6, which we think are all relevant. Of particular importance is the Winnicott reference (Winnicott, 1960), the source of the remarkable claim that “there is no such thing as an infant,” a claim intended to focus attention on the dyad as opposed to the independent motivations of infants in a most extreme way. We have added a variety of references on the issue, and we have taken the suggestion and done a Pubmed literature search, a description of which is detailed in the new SI.**

**But more importantly we have de-emphasized the implied criticism of the prior literature in the revision and have endeavored to make the rationale more positive in its relation to the achievements of prior work on vocal interaction in infancy.**

**Regarding the literature search in Pubmed, where we focused on abstracts only: The term “infant vocalization protophones” was abandoned in the search (it returned only articles with Oller as co-author). The term “infant vocalization” returned many articles however, but most showed no emphasis in the abstracts on social vs. non-social human infant vocalization, and so were rather irrelevant to the current paper’s rationale. Some articles that occurred, for example, merely reported acoustic data on infant sounds, with no mention in the abstract of either independent or interactive production. Many were about cry only. Perhaps the majority were not about human vocalization at all (birds were a particular focus). We ignored all such articles as well as articles with Oller as a co-author. We examined the abstracts for the first 160 articles returned (dates of the 160 articles ranged from 2020 to 2014); only 18 of them could be judged with moderate certainty (based on the abstract only) regarding an emphasis on social use of human speech-like vocalizations as opposed to endogenous or independent use. None of these 18 appeared to have attempted to address the question of the current paper (actually counting and focusing on a comparison of rates of social and non-social use of infant vocalization), and none seemed to have placed emphasis on independent, endogenous production. 15 showed a clear tendency to focus on the social-interactive use of infant sounds, while 3 described use of infant sounds when infants were not interacting, though consideration of interaction was not excluded in these cases. These results are presented in the new SI, and a mention in the main text points the reader to the SI location.**

On a related note, it would be important to clarify exactly the instructions provided in the MTurk questionnaire; is there a way to be certain that respondents had the same definition of 'social' as your coders? **Instructions to coders were quite complex (training lasted weeks) and could not have been presented practically in an mTurk survey. We have deemphasized the mTurk survey, placing it in SI.** How would someone understand "non-socially directed"? Perhaps to be even more certain, the questionnaire could be repeated with other ages (babies, children, adults), to establish that people are not just answering towards the middle of the scale. **Rather than expanding the survey, we have opted for moving it to the SI, and supplying more extensive description.**

Second, in the discussion, the only interpretation that is put forward is also based on a social/dyadic view of child vocalizations. Why would infant production not be also internally motivated? **You seem to have found a wording in the prior version that inspired a mistaken interpretation of our intent. We interpret the data as indicating that the bulk of infant vocal production is indeed internally motivated. The fact that caregivers react to the sounds by differential investment in different infants does not contradict the internal motivation on the part of the infant to produce the sounds. The infant’s intent appears usually to be endogenously-generated exploration, while the parent’s reaction of differential investment in infants who vocalize at different levels provides the selective force on the infant’s endogenous tendency. We have reworded wherever it seemed possible to clarify this point, and have emphasized it in the Discussion.** One could make the case that crawling, walking, mouthing objects are all also fitness signals; but it seems equally plausible that these are all behaviors exhibited by a young learner who is exploring the physics of this world and his/her control of his/her own body. **We agree that anything the baby does (as well as non-behavioral features such as skin color) can constitute a fitness indicator, a point that we have made in prior publications. The novel idea (first expressed independently at about the same time by J. L. Locke) of the origin of language proposal in this article and others we have published is that human vocalization may have come under strong selection pressure as a fitness signal along with a variety of other features that can be read by caregivers as fitness indicators. The pressure on vocalization appears to have been particularly potent and effective in the hominin case because of hominin altriciality. Thus it seems likely that in all primates, movements of the hands, ability to climb, other locomotory skills, and so on are interpreted by conspecific caregivers as fitness signals. Vocalization similar to protophones appears to be largely absent in other primates (with possible exceptions in a few monkey species) and especially in other apes. Our proposal is that hominin vocalization in particular has long been under selection pressure as a fitness signal (and continues to be). These points are addressed in the revised Discussion.** Moreover, it is unclear to what extent the fitness explanation of babbling may not be culturally specific (Brazelton, 1972). **We consider this a considerable gap in the available research, a point we took up at some length in (Oller, Griebel, et al., 2019), and where we highlighted your own work on the Tsimane. Brazelton’s work on the Maya (Brazelton, 1972) preceded any of the significant longitudinal studies of human infant vocal development; he did not have any of the descriptive tools that are used in modern research on vocal development—consequently we are inclined to seek additional research on such cultures, updated to more modern descriptive methods. We are hoping that your work may help in the near future to determine protophone rates in societies of very low technology, and especially where infant-directed speech appears to be low.**

**In the meantime, there are some points worth making about possible cultural and other related differences in protophone rates based on decades of longitudinal work on this topic, results suggesting substantial generality of the human infant tendency to produce massive numbers of protophones. The prior work suggests that protophone rates are (as in the present study) many times higher than in either chimpanzees or bonobos and similar to (although sometimes a bit lower than) the rates reported in the present paper, for infants 1) ranging from very low SES to high SES in the US (only about a 20% reduction has been seen even in very Low SES) (Oller et al., 1995), 2) born prematurely but with no other physical risk factors (Eilers et al., 1993); 3) with a variety of disorders of communication including: Down Syndrome (Lynch et al., 1995), Autism (Patten et al., 2014), Fragile X syndrome (Belardi et al., 2017), and perhaps most surprisingly, profound bilateral congenital deafness (Nathani Iyer & Oller, 2008). Furthermore subsidiary analyses we have conducted of data from others of our publications not directed specifically at protophone rates, but including such data, suggest that at least for Spanish-learning and bilingual infants in Miami (Oller & Eilers, 1982), for Lebanese Arabic infants (Farran et al., 2019), and for Taiwanese infants (Mandarin and Southern Min) (Lee et al., 2017) the rates are comparable to those for the English learning infants in the present work and for example in Oller et al. (Oller, Caskey, et al., 2019).**

**It also bears mentioning that a prior study from our laboratory showed a six-fold higher rate of IDS in laboratory recordings compared with randomly sampled all-day recordings where the infant was awake, all based on human coding (see Supplementary material to Oller et al. (Oller, Griebel, et al., 2019)). The same coding showed very little difference between infant protophone rates in the laboratory and in the all-day home recordings, in both cases 4 per min or higher. These results hint to us that the primary driver of infant vocal rates is internal to the infant. But of course much more extensive cross-cultural data (especially in cultures such as the one Brazelton studied) would inform this conjecture substantially.**

On an unrelated note, given that there is so much variation in the duration of sessions, please consider controlling for that in a supplementary analysis (which could be in an appendix). **The duration variation is indeed controlled for in the Generalized Estimating Equations (Liang & Zeger, 1986). This statistical approach is extremely conservative, while robustly accounting for differences in available data across ages, conditions, and infants. We have emphasized this point in the revision.**

DATA AVAILABILITY
The authors state they cannot share the recordings because parents did not okay this. However, the annotations should be shareable since they are fully deidentifiable. At the very least, the authors should share a table like Table 3 but splitting socially directed versus not, gaze-directed or not. **Will do. In OSF.**

MINOR:
- p. 8 "to estimate the percentage of how many of these sounds" were these literally the instructions? Because this language seems confusing to me (either percentage or how many, not "percentage of how many") **Corrected.**

- p. 8 line 166 for a total of 3 judgments or 9 judgments? **Six. The sentence has been reworded in the SI: “Participants estimated a percentage of social and endogenous infant vocalization functions at three ages (3-month-olds, 6-month-olds, and 10-month-olds) for a total of six judgments.”**

- p. 9 What were the attention checks? **Added in SI lines 134-139** Please check order of responses in Table 1 for frequency around children **Corrected, thank you.**

- p. 10-13 There are several mentions of selection from a larger data set (more children, more recordings). Can you please clarify how you made sure these selections were not biased? **Selections were made on the basis of age and gender matching, with approximately the same age recordings across the first year (3, 6, 10 months). We specify that there was no bias in the selection with regard to social directivity (we didn’t know what the outcome would be and coded the data only from the infants selected).**

p. 14 lines 258+ But doesn't this suggest that you may be over-estimating social vocalizations? I'm also uncertain how to interpret the comment on p. 22, line 434-5 **We have adjusted the wording away from “sociality” to “function.” We apologize for the confusion.**

p. 15 line 275-6 Please specify what other contextual factors. Timing is mentioned in the discussion; what else could be used? And for timing, how was timing integrated? **Because these judgments were intuitive, contextual factors varied wildly. We have added examples following Table 4: “gaze direction, gesture, timing with respect to utterances of other speakers, body positioning, distance between speakers, objects in play”. We explain in the revision that the judgments have to be intuitive because the goal is to characterize social directivity as determined by human observation and intuitive judgment—any rigid method would in our opinion be inferior, and this argument is presented in both the main text and the SI. But we do supply the outcome with gaze direction as the sole criterion, and the outcome is similar.**

p. 17 line 321 "and many more" --> ", particularly" (to avoid a reading in which the first half of the sentence refers to younger babies) **wording adjusted, thank you.**

p. 18 line 344 "resulting" --> "remaining" **corrected**

p. 22 line 439 Do you really mean "suspicious"? **changed to “presume”**

Figure captions: please provide short explanations of "interactive" and "socially directed" **added**

# Response to Reviewer 2: Gratier

Reviewer #2: This paper presents data, first, from a survey of public opinion on the social orientation of infant vocalization in the first year of life and, second, from multiple recordings of infants with their parents in a laboratory study at various ages across the first year. The findings show that contrary to both common belief and dominant scientific theory, infant vocalization is more often nonsocial than directed to another person. The view put forth in the discussion section is that the primary function of preverbal vocalization is not to communicate internal states or messages but rather to signal fitness for developmental adaptation and change. **Our portrayal was a little too limiting, and we have tried to be more clear in the revision. The (illocutionary) function (from the infant’s standpoint), according to our reasoning, is usually exploration and play, perhaps even practice, and on occasion it can be to express various states of emotion; on yet other occasions the illocutionary intent can be to engage in interaction. There need be no fitness signaling intent on the part of the infant, although during interaction it seems likely infants may be motivated also by a desire for bonding, especially as they get older. The fitness signaling “function” is perlocutionary, not corresponding to the infant intent but to the caregiver interpretation of the vocalizations as signals of well-being. We have reasoned since 2005 that a fundamental break in vocal communication of hominins from the great ape background was based on hominin infant vocalization selected for fitness signaling. However, from the infant’s perspective the purpose of the sounds need not have been to signal anything—their actions seem likely to have been motivated primarily (at least early in the first year) by naturally selected internal mechanisms of pleasure associated with vocal exploration in much the same way that other playful or exploratory behaviors are associated with pleasure. But importantly the key change is that the infant sound producing capability, thus selected, was actually free of any particular function, because it had to be possible to produce sounds to serve any function the infant was capable of expressing in any modality or with any action. That is the essence of vocal functional flexibility, a capability upon which we have suggested all other capabilities of language depend (Oller et al., 2013). These points are made briefly in the main text and are explored more extensively in the SI, Appendix D.**

Infant vocalization is thus presented as a residual behavior from the long phylogenetic process of human evolution and as an adaptation to the constraints brought about by altriciality. **We would not call the vocalizations “residual” because that would imply (at least to us) that the pressure that produced the tendency to vocalize so abundantly is now gone. We don’t think so. We presume that even now (perhaps even more now than in the distant past), caregivers (whether consciously or unconsciously) use protophone production as an indicator of infant well-being and as a basis for differential investment. By contrast, crying can be thought of as an appeal for help during distress, and it is clearly an important kind of signal, which has also been interpreted as a fitness signal (Furlow, 1997). But unlike cry, protophones produced in comfort when a parent is nearby but not attending specifically to an infant, can reassure the parent of the infant’s comfort and well-being. Similarly an infant who produces protophones in interaction with the parent can also reassure the parent (probably more potently) of the infant’s fitness. An infant who provides such reassurance commonly may well be at lower risk for neglect or abandonment than the infant who fails to produce such sounds or who cries too much (implying of course that cry can also signal lack of well-being). We are in the process now of studying behavioral measures of adult responses to infant sounds of various sorts (especially during non-interactive events) in an attempt to begin testing the fitness signaling idea, and studies involving physiological measures of adult responses are being developed. There are no doubt hundreds of ways to do this, so we hope others will also take on the challenge or propose other explanations for the massive numbers of protophones produced by human infants.**

Overall the paper is well-written and well-presented, its studies seem rigorously conducted and their findings are reported with sufficient clarity. The main finding, that infants produce many more nonsocial vocalizations both when they are interacting with a parent and when merely in the presence of their parent, is important, novel and challenging. The study provides evidence that, from the cooing to the babbling stages of vocal development, infants mostly partake in vocal play that is not directed toward another person. The evidence is based on naturalistic longitudinal recordings of 6 infants and their parents made by a team of researchers in their University of Memphis laboratory. Although the number of participants is small, the evidence is based on a large tally of individual infant vocalizations, or protophones, identified from multiple segments compiled together for each of two circumstances, interactive and non-interactive. The data is obtained from the audio and video segments. Thus 6649 protophones are categorized by coders based on two types of coding strategies. Each protophone is coded as social or nonsocial, first according to its ‘illocutionary force’ and, second based on whether or not it coincides with gaze oriented to the parent. The first coding strategy is based a subjective appraisal of the context within which the protophone was produced (over a few seconds) and the second is based on a micro-analysis of video footage without sound corresponding to the protophone + or – 50 ms. **We view the subjective judgments of adult human observers to constitute the most relevant possible data regarding infant intent, more so than gaze direction by itself, because there are clear occasions where social directivity occurs in the absence of eye contact (timing is often a key cue in such cases), and there are even occasions where eye contact (or at least gaze in the direction of the parent) occurs but where coders judge the protophone to be not-directed. So the ultimate data are based on intuitive judgments of infant social or non-social intent by coders that balance information from a broad context including gaze direction, relative timing, attention to objects, continuity, and so on. Of course it might be best if we could use the parents of the infants themselves as the judges of social directivity, but the cost of arranging that kind of coding with parents has inhibited us from trying it given the complexity of our laboratory coding technology. The subjective judgments are designed to simulate potential parental interpretations of infant actions. We reason that both parents and potential parents must be good at making such intuitive judgments, since if they were not, they would not be in a position to effectively interpret infant intentions and thus would be unprepared to effectively entrain their infants in vocal communication. We reason there is always Darwinian competition among parents to be effective interpreters of infants’ actions, because those interpretations will determine the success of their parenting and affect their own inclusive fitness (including e.g., their number of grandchildren).**

**So subjective intuitive judgments of human observers based on a balance of factors about infant intents are the gold standard, we think, against which any presumably “objective” measure of those intents should be judged. We have reasoned that the gaze direction analysis by itself, though subsidiary to the balanced intuitive judgments of infant intent, is informative as a cross check. And of course the outcome of the analysis in terms of rates of social directivity (termed “engaged vs. independent” in the revision) of infant vocalizations turns out to be similar whether we use the intuitive illocutionary judgments or the gaze direction judgments.**

Though it is fairly usual in studies using naturalistic data for researchers to reorganize the original data in a way that makes it relevant for the study, the description of the pertinent segments in the methods sections could be made clearer. Firstly, table 2 presents the exact ages at which recordings in both circumstances were made for each of the 6 infants and shows there are 6 recording sessions per infant (with age and condition as within subject factors). The ages range from 3 months to 11;3 months but are grouped into 3 age categories (3, 6 and 10 months). Although there is a clear gap in the data between 7;3 and 9;3 months, there is no obvious reason to distinguish groups between the 3- and 6-month categories. Indeed, much research, mostly by the last author of this paper, has shown fine-grain developmental change in the qualities of infant vocalization (for example, solitary vocal play increasing between 4 and 5 months of age compared to 2 and 3 months of age). I would recommend considering age as continuous, sequential time points rather than as a categorical factor. **Actually my prior work (Oller speaking) on stages of development was not based on quantitative comparisons of those stage differences, except with regard to the shift from precanonical to canonical babbling. The term “vocal play stage” was formulated not by me, but by Betty Stark. My preferred term was and is the “expansion stage” (emphasizing increasing clarity of different identifiable types of vocalizations in the expansion stage rather than a change in function from the prior “primitive articulation stage”) in part because I was not sure how best to quantify any change in the amount of vocal play across stages. I have also been hesitant to draw a formal distinction between play and exploration. But in response to your suggestion, we have conducted an analysis based on age as a continuous variable. And the three authors are now involved in another study with a different set of recordings where intuitive judgments of amounts of vocal play (not distinguished from exploration) at various ages are indeed being analyzed.**

Secondly, table 3 shows how the data had to be recompiled based on identification of segments described as interactive and non interactive circumstances. The authors explain on page 11 that when for example an infant in the non interactive condition (parent talking to another adult), “sought attention from the parents” the segment was re-labelled as ‘interactive’. They also explain that in the interactive sessions the experimenters could engage in conversation with parents and in such a case the segment would also be re-labelled as ‘non interactive’. However, the specific criteria for re-labelling of segments is not clear. What do the authors mean by “sought attention”? Why were parents ever given the opportunity to interrupt interaction with the infant in the “interactive sessions” and in the case of an interruption shouldn’t the whole segment be excluded from analysis? **The Origin of Language Laboratories (OLL) projects directed by Oller since the early 1970’s have always involved fluid naturalistic recordings, only loosely bound by protocols. Because the studies have almost always been longitudinal, we have felt obliged (and our IRBs have required us) to treat the parent as always being in the position to violate protocols of recording in the interest of infant comfort. Consequently, for example, a crying infant often results in an interrupted recording. Similarly an infant bidding for attention during a non-interactive (or Independent, the term used in the revision) session where the parent is presumably supposed to talk with a staff person instead of the baby, often results in an interruption of the Independent protocol. An interactive (or Engaged, the term used in the revision) session can also be interrupted by infant discomfort or fussiness, during which a staff member may enter the room to help, and the parent may engage in conversation with the staff member rather than the infant during the period of adjustment. Another source of such interruption has sometimes been technical glitches, such as microphone failure, which is constantly being monitored by control room staff, who may be required to enter the room and converse with the parent while correcting the technical problem during an Engaged session.**

**As a result the original protocol plans for these recordings were ignored in the analysis with regard to the “engaged” or “independent” sessions. To determine time periods that would be treated as “engaged” or “independent” in the analysis, the sessions were coded moment by moment in a special field based on the parent’s directivity of speech, such that every period of time was assigned to being either engaged (parent attempting to interact with infant) or independent (parent interacting with another person). The determination of infant social directivity (now termed “social” vs. “endogenous”) pertained then to those segments that the coding had assigned to the actual engaged or independent modes of the parent. We have supplied a new table in SI that specifies how much of the original time assigned by protocol to interactive vs. non-interactive resulted in real engaged vs independent activity of the parent.**

By and large I am not convinced by the relevance or usefulness of the survey study. I do not understand its purpose and I feel the paper would be stronger without it. It is fairly obvious that random groups of adults would remember only the vocalizations that are efficient in gaining attention from adults. Even parents involved with their infants on a daily basis often fail to hear the noncry vocalizations they produce. Indeed, even researchers accustomed to careful listening fail to notice some vocalizations based on audio recordings and require visual plots in order to appreciate both quantity and quality of infant sound making (I speak from experience here!). The authors seem to want to show that researchers who have focused their attention on specific brief episodes where infants and adults are socially engaged provide a biased perspective on infant vocalization because they select out most everyday sounds produced by infants. And it is quite convincing enough to state this without recourse to Amazon mTurks. With regard to the design of the survey I am concerned that the wording of the questions entails some bias linked to the use of contrastive questions involving negation (socially-directed vs. NOT directed) rather than two confirmations (e.g. socially-directed and for own enjoyment). **We hope it will be satisfactory from your standpoint to relegate the whole section to SI and to describe the study in more detail, clarifying that the instruction wording did *not* in fact include the biasing phrasing that was suggested by our prior version.**

I am also concerned, at a more theoretical level, that the authors have a rather narrow view of what some researchers mean by the term ‘interaction’ between infants and social partners. **A point well-taken. We have modified our rationale to adjust to acknowledge that the most useful point of our evidence is to help redirect attention to the possible developmental consequences of vocal exploration. We have expanded citations to the literature on interaction and acknowledged more fully the breadth of prior research.**

The authors claim that the large proportion of endogenous, nonsocial or “intrinsically motivated” vocalization they find for all infants at all ages shows that infants are active vocal learners, whereas research showing high rates of social communicative vocalization entails a view of the infant as a passive recipient of vocal input. However, much research on vocal interaction presents a very different perspective, one where the infant on the contrary is involved in a socially-motivated agency which is crucial for any form of interpersonal coordination, intersubjectivity or attunement (see research and positions of Trevarthen, Beebe, Stern). The term “intrinsic motivation” has in fact been used in infant development studies for decades by Trevarthen to refer to the infant’s innate motives for social engagement with other persons. **Again, points well-taken. Infant interactive vocalization is not necessarily passive, and indeed appears to be very active. We agree that it is best to assume the infant is an agent in solitary vocal exploration AND in interaction. Our prior phrasing was not intended to imply that the infant in interaction was purely passive, but rather that the history of behaviorism has, we think, left residues that do not *encourage* thinking of the infant as active to the full extent that seems justified, especially when the infant’s vocalizations are not socially directed. Of course, you are correct that many modern developmentalists do not appear to have been caught up in the legacy of radical behaviorism, and our rephrasings attempt to account for that fact.**

The view that infants learn through active solitary exploration is rooted in a Piagetian approach and has come under serious criticism in infant psychology. **No attempt here to defend Piaget. I (Oller again) do not think I have been deeply influenced by him, although our rewriting includes citations to Piaget. But the question of the extent to which active vocal exploration helps to produce learning of new vocal categories is surely still open. And the fact that human infants produce on the order of 3500 protophones daily raises the question of what effects that extensive exercise has on vocal capability, especially given the present results suggesting the great bulk of the vocalizations are produced in the absence of social intent. We are not inclined to insist that there is a major effect of such exercise (research on tracheostomized infants inspires caution about the practice interpretation, see e.g., (Kraemer et al., 2005; Locke & Pearson, 1990), but we do contend that our new evidence suggests future research should address the possibility that protophone production does at least to some extent constitute practice yielding greater vocal control.**

There has recently been renewed interest in the role of social interaction involving contingent vocalization between adults and infants (such as reflected in studies by Goldstein and collaborators, Hirsh-Pasek and Golinkoff or Pat Kuhl and others) for language acquisition. However, the emphasis of these researchers on social contingency entails a view of interaction as a mechanistic social feedback loop where infants learn responses to cues. Careful descriptive and data-drive research on selected episodes of attentive adult-infant engagement has shown that infants are remarkably active when engaged with another person and explore more varied sound forms when scaffolded by imitative vocalization of their parent (Gratier & Devouche, 2011). It does not seem in any way incompatible that infants may learn both though interactively scaffolded vocal expression and through active solitary exploration of sound-making. In this sense, this study indeed provides invaluable insight into the extent to which infants may engage in vocal play that is not directed to others. But perhaps here quantity is not the crux of the matter, perhaps those brief moments of coordinated engagement between parents or other adults and infants provide as much (or more) developmental gain in less time than longer periods of endogenous self-motivated sound making. **To this point, our response is a change in the focus of the rationale. Rather than presenting the rationale as an indictment of the field (as it may have seemed in the previous version) for focus on interaction (which indeed has been the primary focus), we have refocused to emphasize the importance of *also* considering the possible role of endogenous, solitary vocalization in development. We’ve added citations to help.**

It is indeed intriguing that the authors find that most protophones produced even in the interactive circumstances are nonsocial. The authors assume that a vocalization is social only if it is associated with gaze or other social cues. However, the relative timing of vocalization and of other social cues merit perhaps more detailed analysis than is provided by the coding in this paper. It is plausible that infants rely on multi-modal timing so that they may gaze or smile at a partner not while they vocalize but shortly before or after vocalization. **We think you may have misunderstood the coding. It was designed precisely to account for such possibilities. We’ve tried to make this point more clear in the revision. The coding included a full viewing/listening of every 20 min segment before beginning any coding. Then coding was conducted from start to finish of the segment in such a way that each infant utterance was judged intuitively for social or endogenous intent based on all prior events in the session. All intuitively relevant cues (including timing) were taken into account, and sometimes the timing cues were taken to indicate an utterance was social even in the absence of parent-directed gaze. Thus the factors taken into account included relative timing of infant vocalization with his/her own gaze, facial affect, body movements etc. *as well as* relative timing of infant vocalization with regard to any action or perceived state of the adult. We hold that the intuitive judgments of real, careful observers represent the gold standard for interpreting social intent—any “objective” measure (such as timing in the absence of other cues) would be useful if and only if it corresponded to the intuitive judgments. Consider for example the situation of a parent talking on the telephone while holding an infant who is vocalizing while playing with a toy—timing might sometimes falsely suggest conversational interaction between parent and infant in such a case. We reason that human observers have been evolved to be a good judges of infant intents (poor judges have surely been selected out of the population because their poor parenting skills would have been disadvantageous for their potential offsprings’ reproductivity).**

This finding also suggests that even when parents are attentive to their infants and stimulate their engagement through infant-directed speech, mutual coordinated engagement is rare and brief. But again, however brief, it may be highly adaptive and of great importance to vocal development and to language acquisition and cognitive development. **We agree and have so indicated in the revision.**

The authors ask an important question about their findings. What is the purpose of undirected preverbal vocalization? The explanation provided by the authors (protophone as fitness signal) is thought-provoking but excludes other plausible evolutionary explanations that merit attention. Dean Falk for example has developed a theory that infant-directed speech, evolved in order for parents to maintain continued contact with infants incapable of clinging on to them, gave rise to elaborate vocal interaction paving the way to language. Ellen Dissanayake suggests that the temporal sequencing ability that evolved from multimodal caring interactions between mothers and infants was a crucial adaptation for language and for cultural practices such as ritual and art. It would also be worth discussing to what extent the activity of sound-making is associated with a creative, intelligent and prospective process in infant development rather than acting as mere beeps of a survival instinct. **We have added comments on and references to some of the additional relevant proposals about pressures relevant to the origin of language foundations in ancient hominins in Appendix D. However, we know of no case of a proposal that directly provides an account of the massive numbers of protophones that are produced by human infants—perhaps in part because theorists of language origins aside from ourselves and Locke may have been unaware of the massive numbers and the fact that protophones begin as soon as infants can breathe on their own (Oller, Caskey, et al., 2019). The proposals of Falk (Falk, 2004) and Dissanayake (Dissanayake, 1992), for example, while clearly of interest in the totality of the origin of language, do not (as far as we can tell) address how infant vocal activity would have originated, but rather how mature hominins might have used vocalization (in singing, in IDS). These proposals are inconsistent with the evo-devo principle that natural selection targets developmental processes, selecting for capabilities that begin early in development and then self-organize across maturation and learning to yield new capabilities and functions. Our reading of Falk and Dissayanake and many others (e.g., (Deacon, 1997; Dunbar, 1996; Fitch, 2000; Gärdenfors, 2004; Kirby, 2001; Sinha, 2004) has left the impression that all are relevant to vocal language evolution, but only *after* vocal flexibility has been established. With vocal flexibility in place, singing and IDS (in mature individuals having profited from the experience of flexible vocal activity in infancy and childhood) have a foundation upon which to be built. This sort of scenario of sequences of evolution is consistent with evo-devo reasoning.**

Finally, it is not clear how the fitness signaling function of protophone production prepares infants for the major incremental shifts involved in learning to produce language. **The reasoning we have presented in no way rules out important effects of the production of early protophones in vocal category formation, in tuning the vocal motoric system generally, in forming foundations for the major shift into canonical babbling, and in preparing the infant for elaborate interactive vocalization. All of these are foundations for further developments that we presume to involve culturally-determined pressures that come to bear as the new capacities develop. Fitness signaling surely continues to play a role even in modern adulthood (see e.g., (Miller, 2000), but a wide variety of additional factors surely come into play.**

**Our proposal, elaborated extensively elsewhere (Griebel et al., 2016; Griebel & Oller, 2008; Oller, 2000; Oller et al., 2016; Oller & Griebel, 2005) has never suggested that protophone production by itself accounts for language. The suggestion is that protophone production reveals an infant capacity that is *foundational for additional steps* of development that are clearly also critical for language. The additional steps are hypothesized explicitly in the natural logic described in detail in several of our prior publications, a few of which are cited above (especially (Oller et al., 2016)). The revision attempts briefly to make this point clear.**

In conclusion, this paper provides exciting and thought-provoking findings that deserve to be published and that should stimulate further research on the important questions it raises. My main recommendation is to discard or minimize the opinion survey and to provide some clarification of the selection criteria of the coded segments. I also hope some of the points I have raised regarding the question of agency and vocal learning in social engagement might make their way into the discussion section.

6. PLOS authors have the option to publish the peer review history of their article ([what does this mean?](https://journals.plos.org/plosone/s/editorial-and-peer-review-process#loc-peer-review-history)). If published, this will include your full peer review and any attached files. **We are in agreement with including the peer review and responses as accompaniments to the published article.**

If you choose “no”, your identity will remain anonymous but your review may still be made public.

**Do you want your identity to be public for this peer review?** For information about this choice, including consent withdrawal, please see our [Privacy Policy](https://www.plos.org/privacy-policy).

Reviewer #1: Yes: Alejandrina Cristia

Reviewer #2: Yes: Maya Gratier


[NOTE: If reviewer comments were submitted as an attachment file, they will be attached to this email and accessible via the submission site. Please log into your account, locate the manuscript record, and check for the action link "View Attachments". If this link does not appear, there are no attachment files to be viewed.]

While revising your submission, please upload your figure files to the Preflight Analysis and Conversion Engine (PACE) digital diagnostic tool, <https://pacev2.apexcovantage.com/>. PACE helps ensure that figures meet PLOS requirements. To use PACE, you must first register as a user. Registration is free. Then, login and navigate to the UPLOAD tab, where you will find detailed instructions on how to use the tool. If you encounter any issues or have any questions when using PACE, please email us at [figures@plos.org](mailto:figures@plos.org). Please note that Supporting Information files do not need this step.

# References

Belardi, K., Watson, L. R., Faldowski, R. A., Hazlett, H., Crais, E., Baranek, G. T., McComish, C., Patten, E., & Oller, D. K. (2017). A retrospective video analysis of canonical babbling and volubility in infants with Fragile X syndrome at 9 – 12 months of mge. *Journal of Autism and Developmental Disorders*, *47*(4), 1193–1206. https://doi.org/10.1177/0885066614530659.The

Brazelton, T. B. (1972). Implications of infant development among the Mayan Indians of Mexico. *Human Development*, *15*(2), 90–111. https://doi.org/10.1159/000271232

Deacon, T. W. (1997). *The symbolic species*. W. W. Norton & Co. https://doi.org/10.1093/ww/9780199540884.013.u213400

Dissanayake, E. (1992). *Homo aestheticus: Where art comes from and why*. University of Washington, Seattle. https://doi.org/10.1525/aa.1993.95.1.02a00370

Dunbar, R. M. (1996). *Gossiping, grooming and the evolution of language*. Harvard University Press. https://doi.org/10.1017/s0047404598232040

Eilers, R. E., Oller, D. K., Levine, S., Basinger, D., Lynch, M. P., & Urbano, R. (1993). The role of prematurity and socioeconomic status in the onset of canonical babbling in infants. *Infant Behavior and Development*, *16*(3), 297–315. https://doi.org/10.1016/0163-6383(93)80037-9

Falk, D. (2004). Prelinguistic evolution in early hominins: Whence motherese? *Behavioral and Brain Sciences*, *27*, 491–541. https://doi.org/10.1017/s0140525x04000111

Farran, L. K., Yoo, H., Lee, C. C., Bowman, D. D., & Oller, D. K. (2019). Temporal coordination in mother–infant vocal interaction: A cross-cultural comparison. *Frontiers in Psychology*, *10*, 2374. https://doi.org/10.3389/fpsyg.2019.02374

Fitch, W. T. (2000). The evolution of speech: A comparative review. *Trends in Cognitive Sciences*, *4*, 258–266. https://doi.org/10.1016/s1364-6613(00)01494-7

Furlow, F. B. (1997). Human neonatal cry quality as an honest signal of fitness. *Evolution and Human Behavior*, *18*(3), 175–193. https://doi.org/10.1016/S1090-5138(97)00006-8

Gärdenfors, P. (2004). Cooperation and the evolution of symbolic communication. In D. K. Oller & U. Griebel (Eds.), *The Evolution of Communication Systems: A Comparative Approach* (pp. 237–256). MIT Press. https://doi.org/10.7551/mitpress/2879.001.0001

Griebel, U., & Oller, D. K. (2008). Evolutionary forces favoring contextual flexibility. In D. K. Oller & U. Griebel (Eds.), *Evolution of Communicative Flexibility: Complexity, Creativity and Adaptability in Human and Animal Communication* (pp. 9–40). MIT Press. https://doi.org/10.7551/mitpress/9780262151214.003.0002

Griebel, U., Pepperberg, I. M., & Oller, D. K. (2016). Developmental plasticity and language: A comparative perspective. *Topics in Cognitive Science*, *8*(2), 435–445. https://doi.org/10.1111/tops.12200

Kirby, S. (2001). Spontaneous evolution of linguistic structure - An iterated learning model of the emergence of regularity and irregularity. *IEEE Transactions on Evolutionary Computation*, *5*(2), 102–110. https://doi.org/10.1109/4235.918430

Kraemer, R., Plante, E., & Green, G. E. (2005). Changes in speech and language development of a young child after decannulation. *Journal of Communication Disorders*, *38*(5), 349–358. https://doi.org/10.1016/j.jcomdis.2005.01.002

Lee, C. C., Jhang, Y., Chen, L. M., Relyea, G., & Oller, D. K. (2017). Subtlety of ambient-language effects in babbling: A study of english- and chinese-learning infants at 8, 10, and 12 months. *Language Learning and Development*, *13*(1), 100–126. https://doi.org/10.1080/15475441.2016.1180983

Liang, K.-Y., & Zeger, S. L. (1986). Longitudinal data analysis using generalized linear models. In *Biometrika* (Vol. 73, Issue 1, pp. 13–22).

Locke, J. L., & Pearson, D. M. (1990). Linguistic significance of babbling: Evidence from a tracheostomized infant. *Journal of Child Language*, *17*(1), 1–16. https://doi.org/10.1017/S0305000900013076

Lynch, M. P., Oller, D. K., Steffens, M. L., Levine, S. L., Basinger, D. L., & Umbel, V. (1995). Onset of speech-like vocalizations in infants with Down syndrome. *American Journal of Mental Retardation*, *100*(1), 68–86.

Miller, G. (2000). Sexual selection for indicators of intelligence. *Novartis Foundation Symposium*, *233*, 260–270. https://doi.org/10.1002/0470870850.ch16

Nathani Iyer, S., & Oller, D. K. (2008). Prelinguistic vocal development in infants with typical hearing and infants with severe-to-profound hearing loss. *Volta Rev.*, *108*(2), 115–138. https://doi.org/10.1016/j.immuni.2010.12.017.Two-stage

Oller, D. K. (2000). *The emergence of the speech capacity*. Psychology Press.

Oller, D. K., Buder, E. H., Ramsdell, H. L., Warlaumont, A. S., Chorna, L. B., & Bakeman, R. (2013). Functional flexibility of infant vocalization and the emergence of language. *Proceedings of the National Academy of Sciences*, *110*(16), 6318–6323. https://doi.org/10.1073/pnas.1300337110

Oller, D. K., Caskey, M., Yoo, H., Bene, E. R., Jhang, Y., Lee, C. C. C.-C., Bowman, D. D. A., Long, H. L., Buder, E. H., & Vohr, B. (2019). Preterm and full term infant vocalization and the origin of language. *Scientific Reports*, *9*, 14734. https://doi.org/10.1038/s41598-019-51352-0

Oller, D. K., & Eilers, R. E. (1982). *Similarity of babbling in Spanish-and English-learning babies* (Vol. 9).

Oller, D. K., Eilers, R. E., Basinger, D., Steffens, M. L., & Urbano, R. (1995). Extreme poverty and the development of precursors to the speech capacity. *First Language*, *15*(44), 167–187.

Oller, D. K., & Griebel, U. (2005). Contextual freedom in human infant vocalization and the evolution of language. In R. L. Burgess & K. MacDonald (Eds.), *Evolutionary Perspectives on Human Development* (pp. 135–166). SAGE Publications. https://doi.org/10.4135/9781452233574.n5

Oller, D. K., Griebel, U., Iyer, S. N., Jhang, Y., Warlaumont, A. S., Dale, R., & Call, J. (2019). Language origins viewed in spontaneous and interactive vocal rates of human and bonobo infants. *Frontiers in Psychology*, *10*, 729. https://doi.org/10.3389/fpsyg.2019.00729

Oller, D. K., Griebel, U., & Warlaumont, A. S. (2016). Vocal development as a guide to modeling the evolution of language. In W. Gray, D. K. Oller, R. Dale, & U. Griebel (Eds.), *Topics in cognitive science (topiCS), Special Issue: New Frontiers in Language Evolution and Development* (Vol. 8, pp. 382–392). https://doi.org/10.1111/tops.12198

Patten, E., Belardi, K., Baranek, G. T., Watson, L. R., Labban, J. D., & Oller, D. K. (2014). Vocal patterns in infants with autism spectrum disorder: Canonical babbling status and vocalization frequency. *Journal of Autism and Developmental Disorders*, 1–16. https://doi.org/10.1007/s10803-014-2047-4

Sinha, C. (2004). The evolution of language: From signals to symbols to system. In D. K. Oller & U. Griebel (Eds.), *The Evolution of Communication Systems: A Comparative Approach* (pp. 217–235). MIT Press.

Winnicott, D. W. (1960). The theory of the parent-infant relationship. *The International Journal of Psychoanalysis*, *41*, 585–595.
