# Supplementary material for: Social and endogenous infant vocalizations
Source: PLoS One. 2020 Aug 5;15(8):e0224956. doi: 10.1371/journal.pone.0224956 (PMC7406057; doi:10.1371/journal.pone.0224956)
Supplement: Attachment — Submitted filename: Response to Reviewers -2.docx [file pone.0224956.s003.docx]

Social and endogenous infant vocalizations

Long, H. L., Bowman, D., Yoo, J., Burkhardt-Reed, M. M., Bene, E. R., & Oller, D. K.

**Rebuttal letter: Response to reviewers**

Links for each reviewer:

Response to Editor…2

Response to Reviewer 1: Cristia…3

Response to Reviewer 2: Gratier…4

# Response to Editor

PONE-D-19-29493R1
Social and endogenous infant vocalizations
PLOS ONE

Dear Dr. Long,

Thank you for submitting your manuscript to PLOS ONE. I have now received both reviews on your manuscript. I support the reviewers' view that the manuscript has improved and meets the requirements for publication.

I am giving a decision of Minor Revision because the data can still not be accessed on the OSF website. Both myself and Alejandrina Cristia attempted to visit the page and it was restricted. I agree with the Reviewer that it is good to have the data checked by an external person. Also the reviewer suggested a final proofread for typos. **The manuscript and supplementary have been reviewed and edited for typos and errors.**

Could you please make the OSF website public and communicate this to me together with a submitted proofread manuscript by Aug 14 2020 11:59PM ? If you will need more time than this, please reply to this message or contact the journal office at [plosone@plos.org](mailto:plosone@plos.org). When you're ready to submit your revision, log on to <https://www.editorialmanager.com/pone/> and select the 'Submissions Needing Revision' folder to locate your manuscript file. **The OSF website is now made public.**

Please include the following items when submitting your revised manuscript:

- A rebuttal letter that responds to each point raised by the academic editor and reviewer(s). You should upload this letter as a separate file labeled 'Response to Reviewers'.
- A marked-up copy of your manuscript that highlights changes made to the original version. You should upload this as a separate file labeled 'Revised Manuscript with Track Changes'.
- An unmarked version of your revised paper without tracked changes. You should upload this as a separate file labeled 'Manuscript'.

If you would like to make changes to your financial disclosure, please include your updated statement in your cover letter. Guidelines for resubmitting your figure files are available below the reviewer comments at the end of this letter.

If applicable, we recommend that you deposit your laboratory protocols in protocols.io to enhance the reproducibility of your results. Protocols.io assigns your protocol its own identifier (DOI) so that it can be cited independently in the future. For instructions see: <http://journals.plos.org/plosone/s/submission-guidelines#loc-laboratory-protocols> **We have decided to opt out of submitting a laboratory protocol into a repository. Because the coding scheme for each study out of this lab varies and methodologies are refined over time, we are unable to submit a blanket protocol.**

# Response to Reviewer 1: Cristia

Reviewer #1: The revised manuscript is clear, makes good contact with prior literature, and provides all details necessary for a reader to make their own mind regarding the results, while still presenting the authors' preferred interpretations as such.

Please note that the OSF component is not yet public, so I was not able to check the data. I would be happy to do so when the link is rendered public, since it is always useful to have an outsider check that e.g. column names are understandable. **The OSF data is now made public, we apologize for the error.**

Also, please double check the manuscript for typos. The versions that were uploaded were the old one + the one with tracked changes, which I read for my review. So I'm not sure if there may be typos in the final version (without tracked changes). **The manuscript and supplementary have been reviewed and edited for typos and errors.**

# Response to Reviewer 2: Gratier

No reviews/comments for this revision.
